# Lessons learned from respondent-driven sampling recruitment in Nairobi: experiences from the field

**DOI:** 10.1186/s13104-016-1965-y

**Published:** 2016-03-11

**Authors:** Jerry Okal, Henry F. Raymond, Waimar Tun, Helgar Musyoki, Sufia Dadabhai, Dita Broz, Joan Nyamu, David Kuria, Nicholas Muraguri, Scott Geibel

**Affiliations:** Population Council, Nairobi, Kenya; San Francisco Department of Public Health, San Francisco, CA USA; Population Council, Washington, DC USA; National AIDS and STD Control Programme, Nairobi, Kenya; University of California, San Francisco, Nairobi, Kenya; Division of Global HIV/AIDS, Centers for Disease Control and Prevention, Atlanta, GA USA; Jhpiego-Kenya, Nairobi, Kenya; Palladium Group, Nairobi, Kenya; Ministry of Health, Nairobi, Kenya

**Keywords:** Men who have sex with men (MSM), Female sex workers (FSW), People who injected drugs (PWID), Respondent-driven sampling (RDS), Field experiences

## Abstract

**Background:**

Respondent-driven sampling (RDS) is used in a variety of settings to study hard-to-reach populations at risk for HIV and sexually transmitted infections. However, practices leading to successful recruitment among diverse populations in low-resource settings are seldom reported. We implemented the first, integrated, bio-behavioural surveillance survey among men who have sex with men, female sex workers and people who injected drugs in Nairobi, Kenya.

**Methods:**

The survey period was June 2010 to March 2011, with a target sample size of 600 participants per key populations. Formative research was initially conducted to assess feasibility of the survey. Weekly monitoring reports of respondent characteristics and recruitment chain graphs from NetDraw illustrated patterns and helped to fill recruitment gaps.

**Results:**

RDS worked well with men who have sex with men and female sex workers with recruitment initiating at a desirable pace that was maintained throughout the survey. Networks of people who injected drugs were well-integrated, but recruitment was slower than the men who have sex with men and female sex workers surveys.

**Conclusion:**

By closely monitoring RDS implementation and conducting formative research, RDS studies can effectively develop and adapt strategies to improve recruitment and improve adherence to the underlying RDS theory and assumptions.

## Background

Population groups such as men who have sex with men, female sex workers and people who inject drugs are considered key populations (KP) at risk for HIV infection worldwide. In addition to being at increased risk for HIV, these populations are typically hidden and hard to reach for surveillance activities because some of their behaviors are stigmatized or illegal. In Kenya, men who have sex with men, female sex workers and people who inject drugs, long distance truck drivers, members of fishing communities and their sex partners are recognized as the most important KP [1]. These population groups are considered to engage frequently in high risk behaviors, such as unprotected anal and/or vaginal intercourse, have concurrent multiple sexual partnerships, and share needles that are likely to result in HIV or sexually transmitted infections (STI) infection [2]. The burden of HIV infection and other adverse sexual and reproductive health outcomes is higher among KP than among the general population. HIV prevalence rates among KP in Kenya are between 18 and 30 % compared to approximately 5.6 % of the general adult population [3].

In Kenya, national HIV prevalence estimates are derived from sentinel surveillance activities among pregnant women attending antenatal clinics and nationally-representative, population-based surveys with HIV testing, including the Kenya Demographic and Health Survey (KDHS) and the Kenya AIDS Indicator Survey (KAIS) [3–6]. While population-based surveys provide useful information about HIV prevalence among the general population, deriving similar information among KP is more challenging [7]. Obtaining a representative sample of KP is difficult because the parameters of their “universe” remain unknown. There are no lists containing their names and their whereabouts are often unknown; thus, obtaining a representative sample poses a considerable challenge to traditional methods of survey research [8]. Moreover, as already stated above stigma and engagement in illegal activities makes these populations difficult to reach.

Available HIV prevalence data among KP in Kenya is mostly derived from snowball sampling, targeted sampling, time-location sampling and from incidence modeling data [3, 5, 9, 10]. Although these methods have been used widely, especially among men who have sex with men and female sex workers, they have their own limitations and are not appropriate for all settings and contexts. Most often data derived from some of these sources may be biased as they are usually obtained purposively rather than randomly, thus making it difficult to produce reliable estimates of HIV prevalence and behaviors. Usually simple random samples of lists or registries of individuals are considered the gold standard with which to obtain robust statistically sound samples. Generating exhaustive lists of KP would be a cumbersome, stigmatizing and expensive process, as men who have sex with men, female sex workers and people who inject drugs are by nature “hard to reach populations” that keep changing locations in response to the environment [11, 12]. Taken together, the challenges in reaching these populations and the desire to obtain more representative samples have stimulated the use of alternative sampling methods such as respondent driven sampling (RDS).

RDS is a method used to draw quasi-probability samples of less visible and hidden populations [13–15]. RDS includes aspects of snowball sampling, such as chain referral sampling, but is designed to reduce two biases generally associated with the peer referral methods. These biases are probability of inclusion (i.e., some individuals or groups know more people than others and are more likely to be included in the sample) and clustering of characteristics (i.e., individuals or groups may have a bias towards recruiting others who are like or unlike themselves). Usually an RDS study begins by recruiting a small number of purposely selected people in the target population to serve as seeds. After participating, the seeds are asked to recruit other people that they know in the target population. Hereafter the sampling continues with current sample members recruiting the next wave of sample members until the desired sample size is reached [13–15]. RDS is based on a number of assumptions. First, RDS assumes that members of the target population are networked in such a way that a given RDS study would, provided enough resources, reach every member of the population of interest in the geographic area of interest. If populations are not networked in this fashion bottlenecks between the separate networks that comprise the larger population can compromise the ability of RDS to generate population parameters for the population as a whole. Secondly, RDS assumes that as recruitment progresses along long chains of peer referral randomness enters into recruitment making participants further away from the starting point of recruitment (by seeds) less likely to be similar to those very starting points.

This paper reports on the use of RDS in integrated bio-behavioural surveillance surveys among men who have sex with men, female sex workers and people who inject drugs in Nairobi, Kenya conducted in 2010–2011. These were the first population-based surveys on HIV and STI among these three population groups in Kenya. The study was conducted by the Population Council in collaboration with the U.S. Centers for Disease Control and Prevention (CDC) and the Kenya National AIDS and STIs Control Programme (NASCOP) with technical assistance provided by the University of California, San Francisco. While there is a bounty of literature that report on results of RDS studies there are few manuscripts that document detailed aspects of implementation of RDS studies. The wide field of researchers using or considering using RDS would benefit from closer description of implementation. Although HIV counseling and testing was offered to participants (using parallel rapid HIV testing), and specimens collected for STI testing, the authors have chosen to focus on operational aspects specific to RDS, rather than study activities (such as study operation procedures, training, sample collection, storage as well as wet run with sample participants) that are common to similar research studies. We discuss the operational issues faced with RDS such as respondent’s socio-demographic characteristics, coupon distribution, recruitment of seeds, respondent’s recruitment, site operations, and the adaptations that were made to study procedures to address challenges and the overall lessons learned.

## Methods

From July 2010 to March 2011, the Population Council in partnership with NASCOP and other partners implemented sequential RDS surveys among men who have sex with men, female sex workers and people who inject drugs, Kenya. Information about study operations was mainly collected by asking recruitment questions to participants who came to claim their recruitment rewards, study records and from accounts of the study investigators. These questions assessed refusals and basic demographic characteristics of those who refused to take the coupon and the reason for their refusal. In addition to the feedback from participants, recruitment was closely monitored and weekly monitoring reports of respondent characteristics and recruitment chain analyzed to illustrate recruitment patterns.

The study clinic was located in a safe, centrally-located, government voluntary counselling and testing (VCT) clinic at Kenyatta National Hospital grounds. There were nine staff members who managed the day-to-day operations of study activities at the clinic. These staff included a study coordinator, coupon manager, data manager, receptionist, nurse interviewers and a driver. Prior to implementation of the study, all the staff members received a 2 week training on research ethics, informed consent, survey operating procedures and on administering the questionnaires using personal digital assistants (PDAs). In addition, the team was taken through an orientation on cultural sensitivity to ensure that they provide a welcoming, judgment-free, and trusting environment to potential participants.

### Formative research and selection of seeds

Before the survey implementation began, formative research that entailed key informant interviews (KII), focus group discussions (FGD), and in-depth interviews (IDI) was conducted with key study stakeholders and representatives of the three KP to assess feasibility and guide implementation of the study. A total of ten KIIs were conducted with government officials and staff of non-governmental organizations (NGO) to provide information about characteristics of these three KP, and to assess the feasibility and acceptability of proposed activities. We also conducted FGDs with five groups of up to 6–12 individuals from each of the three KP groups. The purpose of the FGD was to deepen our knowledge of the population behaviors and provide practical information related to the set-up of the study. Persons who gave input but were not comfortable participating in a group setting or those who were identified during FGDs as good sources of information were offered an in person IDI.

Based on formative research findings, eighteen initial participants also known as “seeds,” were selected to initiate coupon-based recruitment in Nairobi. Seeds are individuals who begin recruitment. Seeds were selected purposively to represent the geographical, occupational, social economic and educational diversity of the target populations. Before selection, specific questions were asked to determine the eligibility of seeds. After obtaining consent, potential seeds among men who have sex with men were asked about engaging in oral or anal sex with a man in the last 6 months. Female sex workers were asked about sex work status (having sex with a man in exchange for money, drugs, goods, or services in the last 3 months), amount paid for sex and locations for meeting clients. People who inject drugs were asked questions about drug use in the past 3 months, type of drugs injected, locations for injecting drugs, and where drugs are bought and their cost. All people who inject drugs were asked to show injection track marks/scarring to confirm injection drug use. Eligible seeds were asked to come to the study center to complete an interviewer-administered computer-assisted personal interview (CAPI) and to have their blood drawn for HIV and STI testing, and a pregnancy test for female participants. A limited number of seeds were selected for each KP group to initiate recruitment and additional seeds added based on the progress of recruitment. Each seed and all study participants were provided a primary incentive of 200 KSH (i.e., $2USD) to participate and a secondary incentive of 200 KSH for each eligible peer recruited and enrolled. Seeds were given 3–5 coupons to distribute to their peers whom they encouraged to visit the study site. Seeds, and subsequently those recruited, were all requested to come back to the facility after 2 weeks to collect results of the blood test and receive cash incentives for each referred peer who qualified for the study. The 2 week period was deemed sufficient to process the blood samples and have those recruited make up their mind and enroll in the study. Seeds would be reimbursed only for recruits who participated successfully in the study (i.e., were eligible and also consented to enroll in the study). Seeds did not receive the full reimbursement for ineligible participant (s). In the event that participants recruited by seeds were ineligible, the seeds only received a transport allowance to take them back home. All coupons issued to the recruiter were entered in the RDS Coupon Manager (RDSCM, CDC NHBS V 3.0) system to track distribution, reimbursements and to verify that each coupon was unique and valid.

### Recruitment, interviews and sample collection

When recruits arrived at the study center, they were welcomed by the receptionist who visually verified their coupon before handing the coupons over to the coupon manager. After the coupons were verified using the RDS Coupon Manager software (RDS CM v 3.0 was developed by Cornell University for CDC’s domestic HIV behavioral surveillance program. The software tracks participants through initial coupon validation, unique code generation and allows for study staff to track recruitment payments. In addition, the software records who received what specific coupon thus providing the link data between recruiter and recruits. Other versions of RDSCM (available online) and excel based systems to track coupons are also employed in RDS studies), the coupon manager administered a face-to-face screening interview in CAPI to further confirm eligibility requirements. These questions were the same as those posed to seeds during screening. In addition, the coupon manager answered a subjective question regarding his confidence in the recruit’s eligibility; this question and the response by the coupon manager was not visible to the recruit. The handheld computer then informed the coupon manager if the person was eligible or not eligible. Once the recruit was deemed eligible by the coupon manager, each participant was taken through the informed consent process and if the recruit understood and agreed to all elements of the study they were asked to sign a copy and retain another copy of the consent form. If a participant refused to consent, the coupon manager documented the refusal and terminated recruitment procedures. Signed consent forms were stored in a secure location and the consent form information was not linked to survey results. After obtaining consent, the coupon manager used computer software to generate a unique number code based on an algorithm generated by an image of the participant’s fingerprint (actual fingerprint images were not retained) to prevent multiple enrollments. If the biometric device was unable to create a unique number code, a manual system for developing a unique ID was used. The unique ID was comprised of the first two letters of province of birth (or country of birth for non-Kenyans), two numeric digits for month of birth, two numeric digits for date of birth, first two letters of ethnic group, and first two letters of participants sex. These unique codes and unique IDs also served to ensure that HIV and STI testing results were given to the correct participant and to manage compensating participants. No names or other identifying information were collected in the unique ID. If the unique biometrically-derived number or unique ID already appeared in the database, the participant was made ineligible due to high suspicion of prior participation. Additionally, confirmation of the unique code or unique ID in the study database during the second visit helped ensure that the right person received test results and compensation. The unique ID was a serial number that was numbered/given in order of participation and pre-printed in the recruitment coupons. The unique ID of the seed and non-seed recruits was matched with coupons of those recruited. Matching the coupon numbers helped the study team track the recruitment process by determining who recruited who.

Upon completion of these steps, the coupon manager took the participant to a nurse counselor to be interviewed. The interview had five parts: a 45 min behavioral questionnaire, administered by the nurse counselor (who served as interviewer); HIV pre-test counseling and a rapid HIV test; specimen collection (blood, rectal and urine samples); post-test counseling and HIV rapid test results; and referrals and provision of prevention materials. The questionnaire collected information on demographics, alcohol and drug use, sexual risk taking and HIV prevention behaviors, HIV testing history, and experience with violence and discrimination. After the interview, HIV counseling, and sample collection, the coupon manager gave each participant between one and three coupons to recruit their peers into the study.

The coupon manager trained participants on how to recruit their peers, with specific emphasis placed on recruitment within their social networks. Eligibility and screening criteria for all three populations surveyed included being 18 years or older and a resident of Nairobi or surrounding communities. Men who have sex with men also had to report having had anal or oral sex with a man within the last 6 months. Female sex workers had to report selling sex for money, drugs, or goods to a man at least once in the past 3 months. People Who Inject Drugs participants had to have used needles for injection drug use in the past 3 months and had demonstrable track marks (needle injection marking/scarring). As already noted, study participants could have, however, lived in the larger Nairobi metropolis, as long as they were members of the target populations.

### Coupon distribution and monitoring

Coupons were pre-printed and issuance and receipt of coupons was monitored using RDSCM. Men who have sex with men were given three coupons each while the number of coupons given to female sex workers and people who inject drugs was variable. Female sex workers were initially given three coupons each, which was eventually reduced to one and then no coupons due to overwhelmingly high response rates from female sex workers. People who inject drugs were initially given two coupons each and as recruitment progressed, the number increased to three due to the slow recruitment among people who inject drugs. Coupons contained the following information: a unique ID number (coupon number), survey name (the target group and exact purpose of the study was not mentioned to protect potential participants from inadvertently being identified as a member of a KP), interview site address, telephone number of interview site, email address of the project, days and hours of site operation, activation and expiry date for the coupon, proposed date and time for first visit, and date when the coupon was redeemed and retained by the coupon manager.

The information in the RDSCM database enabled us to link recruiters and recruits and to determine when respondents should be paid and how much. This software also helped to prevent the redemption of duplicated coupons because the database would not accept duplicate coupon numbers. Weekly monitoring reports of respondent characteristics and recruitment chain graphs from NetDraw v 2.075 (Analytic Technologies) were prepared by the data manager, used to illustrate recruitment patterns and helped study staff make adjustments to coupon distribution to manage recruitment and maximize representation.

Once each group approached the planned sample size, no coupons were given out to remaining recruits. Coupon distribution was stopped approximately 2 weeks prior to the end date of each survey. The last 2 weeks of each data collection period was set aside to allow the remainder of participants to come in for their second visit (to collect test results and compensation for successful recruitment).

### Ethical approval

The study protocol was reviewed and approved by the Kenyatta National Hospital Ethics and Research Committee, the Population Council Institutional Review Board, and the Associate Director of Science in the Center for Global Health at the CDC.

## Results

### Recruitment of seeds

In this study, as in most RDS studies, purposefully selected seeds were used to begin recruitment. Seeds were selected by the study investigators based on results from formative assessments. Selected seeds met the respective eligibility criteria for the study and were given coupons and instructions for peer recruitment. Although not a requirement of RDS, the seeds were recruited to resemble different demographics (education, socioeconomic status, age, place of residence, and ethnicity) within the sampling area. Seeds were oriented and motivated at the survey start to promote a feeling of survey ownership and enthusiasm about the project. Recruitment was initiated with a small number of seeds to assess patterns of recruitment and additional seeds were planted when recruitment was slower than anticipated or too many waves died out. In total, seventeen seeds were selected based on results from formative assessments men who have sex with men, n = 6; female sex workers, n = 5; people who inject drugs, n = 6). The percentages presented below were RDSAT weighted estimates using RDS Analysis Tool (RDSAT), which weights estimates to account for participant network size and homophily.

### Men who have sex with men

**Fig. 1:**
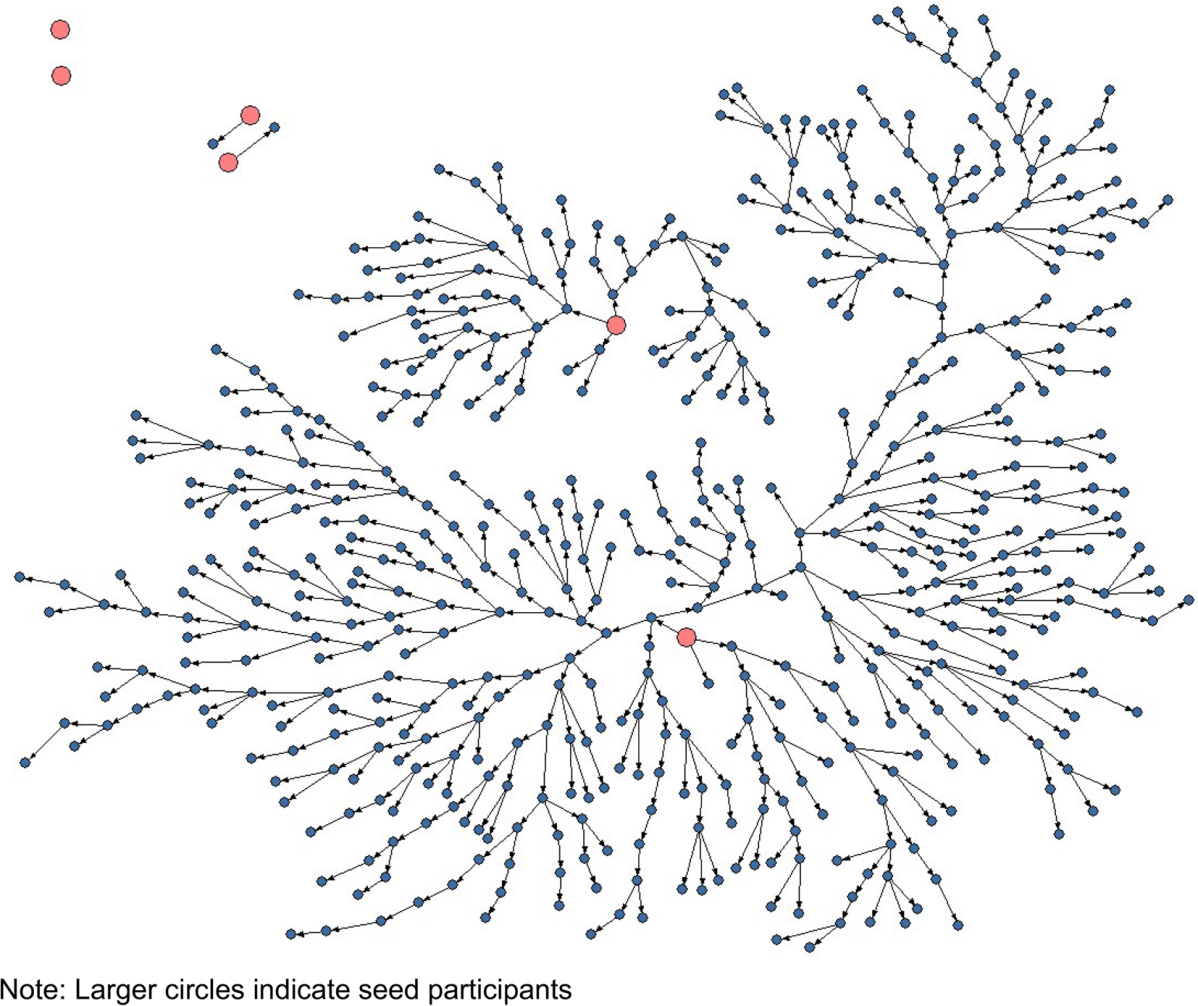
Recruitment of men who have sex with men in Nairobi (N = 569 including seeds). Recruitment was initiated by six seeds who issued 1478 coupons, 691 were returned and out of these 563 met eligibility criteria for study participation and were enrolled in the study. The waves of recruitment per seed ranged from 1 to 23, and the mean network size was 77, while the median was 8 (IQR: 4, 20). *Larger circles* indicate seed participants

The initial aim of the study was to recruit 600 men who have sex with men between July and October 2010. After 3 months, 563 men who have sex with men were enrolled in the study. Out of the 1478 coupons issued, 691 were returned giving a coupon return rate of 46.7 %. Of those who returned a coupon, 563/691 (81.5 %) met eligibility criteria for study participation and were enrolled in the study. The waves of recruitment per seed ranged from 1 to 23, and the mean network size was 77, while the median was 8 (IQR: 4, 20). Out of the six men who have sex with men seeds, two were the starting points for most of the recruitment that nearly achieved the target sample size. These two seeds were diverse by age (younger versus older men who have sex with men) and marital status (married versus unmarried men who have sex with men). Two seeds failed to recruit any of their peers (see Fig. 1).

Recruitment of men who have sex with men started out quite rapidly in the first 2 weeks but slowed down 2 weeks later before resuming a more rapid pace. The slump in recruitment coincided with the annual Muslim observance of Ramadan but it was not clear to the investigators whether the annual religious festival was directly responsible for the drop in recruitment. A review of initial recruitment patterns did not show any particular bias towards Muslim recruits allaying fears that Ramadan contributed to the slow recruitment. Moreover, the final men who have sex with men data showed that the ethnic representation of men who have sex with men mirrored the population living in Nairobi where 11 % are Muslim [5]. This suggests that there was no relation between the slump in recruitment and observance of Ramadan. The slow recruitment prompted the study investigators to plant additional seeds however these additional seeds were not productive. Figure 1 shows the recruitment patterns among men who have sex with men.

Overall, the recruitment patterns show that RDS worked well with men who have sex with men as diverse groups are well-networked. The population is integrated by several characteristics such as age, education, marital status and ethnicity. Men of different ages, levels of education, marital status (those that are single or married to women) and of different ethnic backgrounds successfully recruited among their peers within Nairobi and adjacent areas. However, the study was not successful in recruiting high socio-economic status men who have sex with men especially those drawn from the African and Kenyan Asian community. Before implementation of the survey, we had meetings with members of these groups, provided them with information about the study and encouraged them to participate in the study by planting two seeds within the community but the response was poor. We did not reach out to white men who have sex with men either although anecdotal evidence suggests their presence in Nairobi.

The median age of recruited men who have sex with men was 28 years old (IQR 24–35 years). Over half of the men in Nairobi were <30 years with an estimated one-third of men who have sex with men aged 18–24 years (33.6 %) and one-quarter aged 25–29 years (26.8 %) [16, 17]. More than 80 % of men who have sex with men in Nairobi were estimated to have either primary or secondary education while about 14 % have a tertiary education. About 80 % of men who have sex with men were estimated to be Christians, 14.9 % Muslims and remaining 6.7 % were of other religious backgrounds (including Hindu) or not subscribing to any particular religious faith. Majority of respondents were of Kikuyu ethnic background (41.8 %) followed by Luo (16.6 %) and Luhya (14.7 %). The ethnic distribution of men who have sex with men is largely similar to the ethnic distribution of the overall population living in Nairobi [5]. About 60.3 % of men who have sex with men were single and had never been married. More than a quarter of men who have sex with men had ever been married to women and 13.0 % were still currently married to a woman. Of all men who have sex with men, about a quarter were living with a male sex partner and 12.5 % were living with a female sexual partner.

### Female sex workers

From November 2010 to January 2011, 596 female sex workers were recruited into the survey. Five seeds were selected based on results from formative assessments. Of the five seeds only one seed was unable to recruit her peers. Similar to the men who have sex with men survey, seeds were selected based on socio-demographic characteristics such as geographical, occupational (brothel versus street based etc.), social economic and educational diversity of the target population. Four seeds were productive and helped initiate the distribution of 1219 coupons, of which 632 were returned giving a coupon return rate of 52.1 %. Of those that returned the coupon, 596/632 (94.3 %) met eligibility criteria and were enrolled in the study. The median network size was 8 female sex workers (IQR 4–20) and the waves of recruitment per seed ranged from 18 to 62.

Recruitment of female sex workers started off intensely and on average 15–20 participants visited the study site on a daily basis. This high number of participants was not anticipated and stretched study staff capacity and resources to the limit. Therefore study staff attended to participants from very early in the morning till late in the evening. In order to control the client flow and to better manage participants the study team decided to reduce the number of coupons issued per person when it became apparent that there were an overwhelming number of participants coming to the study site. As such, issuance of coupons was reduced from three to one. Issuance of coupons was controlled to balance geographical distribution of participants from other parts of Nairobi since it was evident that recruitment was skewed towards the eastern part of Nairobi. Additionally, coupons were assigned specific validity dates and participants instructed to advise their recruits to adhere to the dates. These measures somewhat helped control the flow of participants nonetheless a large number of participants (approximately 15) arrived at the study site every day. Even though we were able to see a high number of study participants every day and attained the target sample in relatively short period of time, very few high socio-economic status (SES) women and brothel-based sex workers were recruited in the study. This is despite concerted efforts by the study investigators to seek opinions of high SES women and those working in brothels with a view of enrolling them in the study.

Other than the high participant flow, the female sex workers survey presented another unique challenge that required urgent action. Nearly 30 % of participants came to the study site accompanied by their young children something the study team did not expect but had to deal with since it could affect whether a participant enrolled. In order to accommodate such participants, we purchased and stocked baby milk to feed children who accompanied their mothers to the site. On site provision of baby milk worked well as mothers did not have to worry about feeding their children. Provision of baby milk helped participants patiently wait for their turn to be enrolled into the study. Similarly, the study staff helped with holding children while the mother self-administered the vaginal swab or used the ladies room to collect their urine sample for the pregnancy test. Identifying and addressing these needs meant that the study was able to recruit a substantial number of female sex workers with children.

**Fig. 2:**
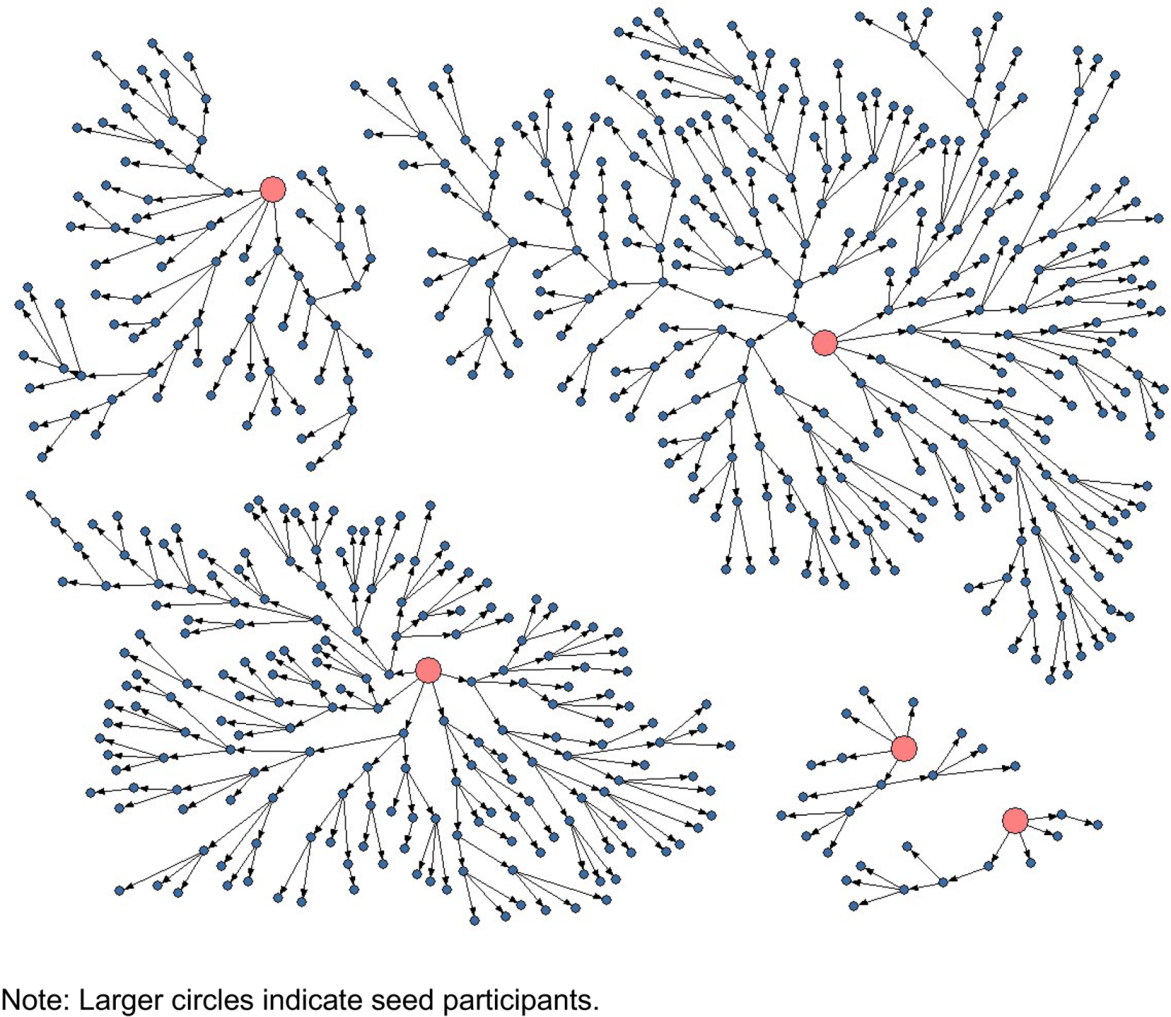
Recruitment of female sex workers, Nairobi 2010–2011 (n = 601 including seeds). Five seeds initiated recruitment and issued 1219 coupons, 632 were returned of which 596 (94.3 %) met eligibility criteria and were enrolled in the study. The median network size was 8 female sex workers (IQR 4–20). The waves of recruitment per seed ranged from 18 to 62. *Larger circles* indicate seed participants

The implication of the rapid client flow was that recruitment of female sex workers was completed within a short period of time compared to men who have sex with men and people who inject drugs surveys. Similarly, female sex workers achieved sample size and reached crude sample stability before networks “branched out” to types of female sex workers that were identified during the formative research. An analysis of female sex workers recruitment patterns in Nairobi also showed that female sex workers have large networks but appear to be not well-connected across neighborhoods. For instance, female sex workers living in the eastern part of the city only recruited their peers from the same locality or neighborhood—thus female sex workers had strongly homogenous recruitment within subgroups. That is peers recruited peers who were of their own social class and lived in their own neighborhood. This potentially explains why large numbers of participants (approximately 15–20 per day) presented themselves every day at the study site something that might be attributed to peer–peer familiarity. As noted before, another emerging pattern was that recruitment of female sex workers was skewed towards the eastern parts of Nairobi. Although female sex workers were recruited from all the eight constituencies of Nairobi; recruitment of female sex workers was concentrated in the eastern parts of Nairobi especially in Kamukunji and Kasarani areas. We were unable to adequately survey the entire city given time and sample size limitations. Figure 2 illustrates recruitment of female sex workers in Nairobi.

The majority of female sex workers in Nairobi were of Kenyan origin (97.7 %) with only 2.3 % from different nationalities (mainly resident of communities in the neighboring East African countries) [17]. The median age of female sex workers was 30 years (IQR 24–38), with the majority (53.3 %) under age 30 years. Nearly all female sex workers (99.2 %) were not currently married although over half (57.4 %) have been previously married. A large proportion of female sex workers had no education or attained an incomplete primary school education (44.0 %). An additional 30.5 % of female sex workers completed primary school, 13.0 % attended some secondary school, and 11.2 % completed secondary school. The majority of female sex workers (71.7 %) owned a mobile phone. For most female sex workers (81.4 %), sex work was their primary source of income and the vast majority (94.7 %) of female sex workers provided financial support to someone else. Female sex workers supported a median of three people (IQR 2–4). More than three-quarters of female sex workers (80.5 %) live with their children. The median number of female sex workers children is two (IQR 1–3).

### People who inject drugs

**Fig. 3:**
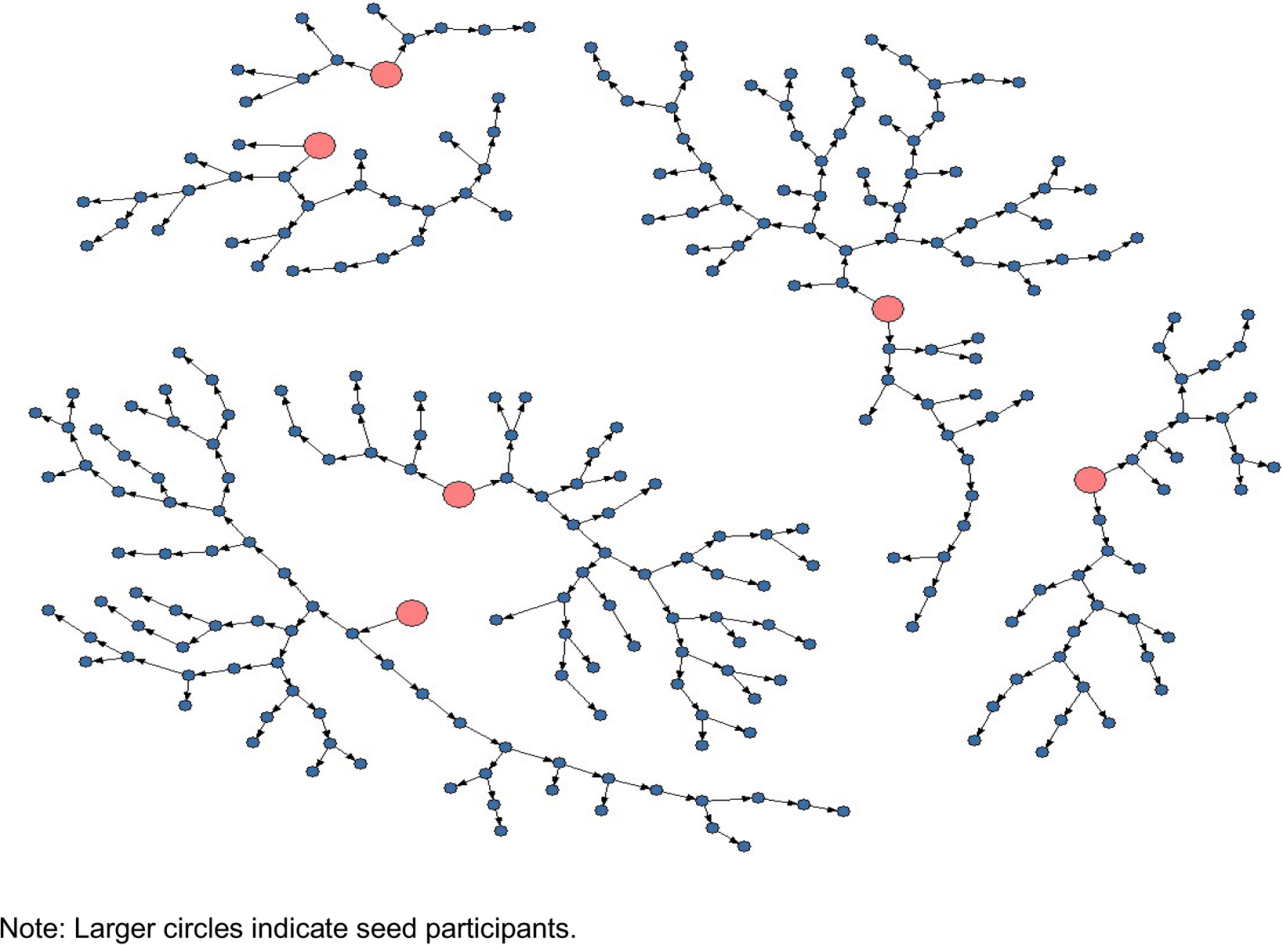
Recruitment of people who inject drugs in Nairobi, 2011 (n = 275 including seeds). Recruitment of IDUs was initiated by six seeds who distributed 473 coupons, 352 were returned to the study site and a total of 269 individuals met eligibility criteria for study participation and were enrolled (76.4 % eligibility rate of returned coupons). *Larger circles* indicate seed participants

The people who inject drugs survey was initiated from January to March 2011 and recruitment of people who inject drugs was initiated by six seed participants who were selected based on results from formative assessment. In total 473 coupons were distributed over 3 months and 352 were returned to the study site giving a coupon return rate of 74.4 %. Of those who returned a coupon, a total of 269 individuals met eligibility criteria for study participation and were enrolled (76.4 % eligibility rate of returned coupons). A large proportion of participants (21 %; n = 78) were found ineligible and were disqualified (31 before screening, 37 after screening) for strong suspicion of not being injecting drug users [18]. Figure 3 shows the recruitment of people who inject drugs in Nairobi.

When compared to the men who have sex with men and female sex workers recruitment, people who inject drugs recruitment was marked by slow flow of participants. As the flow of potential participants continued throughout the survey, the investigators planted additional seeds and provided free transport to encourage recruits to come to the study site. We planted two extra seeds but there was no change in recruitment. With regards to transporting participants, we worked closely with key people who inject drugs stakeholders and outreach workers who informed participants with coupons about the place, time and date of transport pick up. Many people who inject drugs turned up in large numbers at the transport pick up points immediately when they were informed of the service. Essentially the transport pick up points were close to the drug injection areas better known locally as “bases”.

In addition, study staff also had problems screening out non-injecting drug users who were attempting to enroll in the study as people who inject drugs. To ensure that non-eligible participants were not enrolled, the coupon manager and nurse counselors were extra vigilant during participant’s eligibility assessment. The pre-enrollment assessment for people who inject drugs involved asking a set of structured questions on injection drug use and seeing injection track marks. Where there were doubts about an individual’s eligibility, the coupon manager or nurse counselors consulted a team of outreach workers who were on standby (who were also former injecting drug users) to verify participant’s eligibility by asking specific questions related to injection drug use (such as how drugs are prepared and how drugs are injected) and re-examining injection stigmata.

Similar, to men who have sex with men and female sex workers surveys, we were unable to recruit higher socio-economic seeds among people who inject drugs despite the efforts made to recruit them. Those that were approached promised to participate in the study but never showed up.

The median age of participants who inject drugs was 31 years (IQR: 27–37), and nearly three quarters of the participants were aged 30 years or older (64.4 %) [19]. In terms of gender the majority of people who inject drugs were men (92.5 %), with fewer than 10 % women people who inject drugs in Nairobi (7.5 %). Just over half of people who inject drugs had only a primary education or less (51.6 %). While only 16.9 % of people who inject drugs were currently married, over half (58.6 %) had been previously married and about one-quarter (24.4 %) had never been married. It is estimated that over two-thirds of people who inject drugs resided in the eastern side of Nairobi city, namely, Starehe (59.0 %) and Kamukunji (9.7 %) constituencies. Slightly more than 10 % (11.7 %) lived in Dagoreti constituency, which is located on the western side of Nairobi. The remaining 19.6 % were spread over other constituencies and surrounding areas of the capital city. Most people who inject drugs earned money through informal or irregular employment, with only a small proportion (11.5 %) reporting being formally employed. Some participants who inject drugs (18.1 %) earned income through illegal activities, which included stealing money or goods.

Overall in order to prevent double recruits and ensure that the correct participants were reimbursed their transport money a biometric devise was used to record participant’s fingerprints among the three target groups. In the rare instance where this was not feasible a unique ID was used. Among all the target groups fewer than 10 % of the participants required the manual unique ID because the biometric system failed to capture their fingerprints. This occurred mainly because fingerprint reader was unable to capture an image of sufficient quality because the fingerprint was damaged due to involvement in continuous hard labor or smoking.

## Discussion

### Lessons learned from the RDS survey

We learned a number of lessons from implementing RDS studies among men who have sex with men, female sex workers and people who inject drugs in Nairobi, Kenya. Centralized study sites, particularly one where there is substantial other activity can be an advantage for men who have sex with men and female sex workers. The campus area which was near the Nairobi CBD is a busy area and houses the national referral hospital, university campus, doctors’ offices, police station, hostels and residential houses. The busy environment made it convenient for participants to come to the study site unnoticed potentially protecting their privacy. Conversely, while the study site provided a safe and non-judgmental environment for people who inject drugs as well, the centralized location presented difficulties in access for people who inject drugs. Table 1 shows how adapting to practicalities can improve theoretical fit for RDS.

**Table 1:** Practicalities of the study and theoretical fit with RDS

Population	Topic	Issue	Solution	Fit with RDS	Helped success
MSM and FSW	Study site	Need for confidential comfortable acceptable study site within budgetary constraints	“Hide” study site in plain view at a public space that because of extensive foot traffic and nature of environment made KP invisible	One study office reduces potential for duplicate enrollment that is a potential issue with multiple study sites	Contributed to willingness of KP to participate thus obtaining sample size
MSM	High socio-economic status (SES) MSM	Concerted efforts made but it was difficult to recruit high SES MSM and FSW	Conduct rigorous formative assessment to understand the context of high SES MSM Determine disincentives to study participation and mitigate the risk associated with disclosure of sexual orientation/practice among high SES MSM Potentially set up study site in acceptable high end area to serve high SES individuals	Ensures high SES subgroups are recruited	Ensures participation of diverse population sub groups
	Recruitment monitoring	Weekly monitoring of respondent characteristics	Use of NetDraw helped illustrate recruitment patterns	Ensures recruitment flaws are addressed	Helped identify recruitment gaps
IDU	Study site	Accessibility	Provide transport from pick up points in specific neighborhoods	Reduced need for multiple study sites	Increased participation
	Recruitment monitoring	Weekly monitoring of respondent characteristics	Use of NetDraw helped illustrate recruitment patterns	Ensures recruitment gaps are filled	Helped identify flaws recruitment gaps
FSW	Coupons	Too rapid sample accrual overwhelming site capacity	Reduce number of coupons to one per recruiter	Ensures balance to RDS recruitment chains	Better penetration of study population
	High SES FSW and those working in brothels	We made efforts but failed to recruit high SES FSW and those working in brothels	Conduct more rigorous formative assessment to understand the context of high SES FSW and those working in brothels	Ensures high SES subgroups are recruited	Ensures participation of more hidden population groups
	Addressing unique needs of participants	Being sensitive and understanding the needs of participants	Provision of milk to children who accompanied their mothers and helping out with caring for children	Potentially ensured participation of women who had children	Increased participation
	Recruitment monitoring	Weekly monitoring of respondent characteristics	Use of NetDraw helped illustrate recruitment patterns	Ensures recruitment gaps are filled	Ensures coupon issuance was controlled and recruitment gaps are filled

Although our data show that there was a measure of success in penetrating networks of participants who inject drugs, it was apparent that we did not reach our target sample size. Slow recruitment appeared to have been occasioned by loss of coupons due to negligence or due to the psychological effects of the drugs. Our results also show that centralizing the study at the KNH campus somehow limited individual participation. Although during the formative assessment most participants who inject drugs reported that the study site was appropriate and centrally located, informal feedback from some participants indicated that distance to the study site presented difficulties for some of them. Transportation challenges including high transport cost and “competing demands” of sustaining drug use, such as earning money closer to drug markets, likely discouraged some participants from coming to the study site. There were those who lacked transport money to bring them to the site whereas some were actively engaged throughout day looking for money to purchase drugs and would not take out time to come to the study site. These challenges likely reduced motivation to participate in the study. Thus, if we are looking to conduct a similar survey among people who inject drugs we would establish study sites nearer to drug use sites or “bases” as they are locally known. Provision of intensive transport support for future studies is also a feasible strategy but may not be sustainable for ongoing surveillance efforts. Also conducting cultural sensitivity training among field staff is essential to ensure that staff have non-judgmental attitude which enhances participant’s confidence to take part in the study. Similarly, creating strong collaborations with various KP players such as service providers, Ministry of Health and advocates can help forestall any implementation challenges. Strong collaboration with a local organization (s) including their staff during survey implementation helped identify individuals who were not people who inject drugs and prevent them from enrolling in the study, thus increasing internal validity of the survey.

This study also demonstrates the feasibility of fingerprint-based individual identification for RDS in developing countries. Nearly all participants in the study willingly accepted to have their fingerprints collected electronically. This enabled the study team to detect double recruits as well as ensure that registered participants receive their compensation. However, the use of manual unique ID is also necessary in RDS given the difficulties of collecting electronic fingerprint from some individuals. Such difficulties maybe due to physical constraints, such as damaged fingerprints, finger amputations or injuries. In any case, manual IDs may act as back up to the electronic fingerprint in the event that the electronic system fails, participant refuses to provide a fingerprint or the electronic system is unable to capture an image of sufficient quality for successful fingerprint template extraction. In addition, we would recommend implementers to conduct more rigorous formative research to better understand the needs of people who inject drugs in their study sites. In particular, we would recommend seeking to understand what would motivate or de-motivate people who inject drugs from participating in similar surveys especially women and those in the higher SES category. We would also recommend exploring suitability of proposed study site, socio-economic characteristics and network sizes, transport challenges (e.g., use of public transport system and cost) and also determine appropriate incentives. In this study, we provided cash incentives to people who inject drugs based on information gathered from the formative research but our experience suggest that money in itself was not a strong motivator for study participation. Based on our experience, future studies may need to explore the viability of alternative incentives such as offering free health checkups, referral for addiction counseling and treatment, and provision of sterile injection paraphernalia among other incentives.

We recommend recruiting the lowest number of seeds deemed feasible (in our case 6 per group) for the groups of interest and to maximize diverse recruitment changes. Selecting diverse seeds based on geographical locale and SES for highly mobile populations such as female sex workers has potential to penetrate both horizontal and vertical networks [20]. More so, issues such as reducing the number of coupons per recruiter to 1–2 and increasing the target sample size to ≥1000 may need to be properly addressed at the outset in order to manage client flow and produce a more representative sample and more generalizable findings. Conversely, in populations that may be less motivated to participate we would recommend starting recruitment with higher numbers of coupons per person (up to five coupons). Another alternative, however, if the resources permit, is to increase the survey period to allow enough time for coupon distribution and return rate. This will insure that as many coupons as possible are distributed thereby mitigating against loss of coupons and would provide the study more time to penetrate deeper into diverse networks.

Corroborating with lessons learned from other RDS studies [20, 21], future studies may consider using cell phones for screening as cell phones usage continues to grow in sub-Saharan Africa as well as in other parts of the world where RDS studies are being conducted. This would help to ensure that only eligible respondents travel to the study sites, thus, saving on the respondents’ time, transport costs, reducing the cost of the study by cutting down on reimbursements, achieving the target eligible sample size within a relatively shorter period, and managing client flow by minimizing overcrowding at the study site. Phone calls could also be used as a reminder to seeds to distribute coupons and hence may reduce incidents of coupon loss. Finally, getting feedback from participants on potential recruits who refused to take coupons can provide important lessons for RDS and help shape the recruitment process.

## Conclusion

In sum, we learned and recommend that location of the study site, seed selection, and provision of appropriate incentives, providing intensive transport support or otherwise moving the study sites closer to respondents’ meeting or injection (bases) points would motivate recruits and thus enhance the recruitment rate, particularly among people who inject drugs, in future studies. In our study, the survey among people who inject drugs experienced less optimal participation compared with men who have sex with men and female sex workers, we experienced a slow recruitment among people who inject drugs in comparison to recruitment behaviors among men who have sex with men and female sex workers. Using RDS could pose a challenge in environments where people who inject drugs are highly stigmatized, illegalized, and not familiar with research and where they have potentially smaller network sizes. Perhaps complementing findings from RDS with other methods applicable in surveying hard-to-reach populations, such as snowball sampling, capture-recapture, and time-location surveying, could help validate results from RDS and inform planning [22, 23].

Some of the lessons learned about conducting RDS may apply only to Nairobi city. RDS worked well with men who have sex with men and female sex workers populations because many participants were enthusiastic and willing to travel to a central study site to participate in the survey. Although we experienced a few challenges, RDS also worked relatively well with people who inject drugs. More so, drug users in Nairobi are generally understudied population, and still face disproportionate stigma and discrimination, thus, some degree of lack of motivation to participate is expected. In terms of the RDS implementation, we carefully monitored the survey and took necessary measures to ensure data collection was successful. Nonetheless, the lessons learned in Nairobi among the three study populations may be useful to others contemplating implementing RDS studies in other settings. Therefore RDS studies should pay special attention to following elements: location of the study site, seed selection, and provision of appropriate incentives, being aware of the day-to-day challenges and most of all conducting rigorous formative assessment to inform RDS implementation.

In spite of the challenges we experienced, the utility and effectiveness of RDS to recruit hidden population groups cannot be underestimated. Moreover, RDS is still the most suitable method in studying hidden and less visible population groups where robust statistical inference to the population is needed because the peer to peer approach is appealing and confer privacy to group members.

## References

[1] National AIDS Control Council. Kenya National AIDS Strategic Plan 2009/10–2012/13. Final Report. Nairobi; 2009.

[2] Nieburg P, Carty L. HIV prevention among injecting drug users in Kenya and Tanzania (Washington DC: Center for Strategic & International Studies). 2011. http://csis.org/files/publication/110428_Nieburg_HIV_Web.pd.

[3] National AIDS and STI Control Programme, Ministry of Health, Kenya. Kenya aids indicator survey 2012. Final Report. Nairobi; 2013.

[4] Central Bureau of Statistics (CBS) [Kenya], Ministry of Health (MOH) [Kenya] and ORC Macro (2004). Kenya demographic and health survey 2003: key findings.

[5] Kenya National Bureau of Statistics (KNBS) and ICF Macro (2010). Kenya demographic and health survey 2008–2009.

[6] National AIDS and STI Control Programme, Ministry of Health, Kenya. Kenya aids indicator survey 2007. Final Report. Nairobi; 2013.

[7] Githuka G, Hladik W, Mwalili S, Cherutich P, Muthui M, Gitonga J, Maina WK, Kim AA (2014). Populations at increased risk for HIV infection in Kenya: results from a national population-based household survey, 2012. J Acquir Immune Defic Syndr.

[8] Greg S (2008). “They got their program, and I got mine”: a cautionary tale concerning the ethical implications of using respondent-driven sampling to study injection drug users. Int J Drug Policy.

[9] Gelmon L (2009). Kenya HIV prevention response and modes of transmission analysis.

[10] Guows DD (2006). Short term estimates of adult HIV incidence by mode of transmission: Kenya and Thailand as examples. Sex Transm Infect.

[11] Faugier J, Sargeant M. Sampling hard to reach populations. J Adv Nurs. 1997;26(4):790–7. Retrieved from http://www.ncbi.nlm.nih.gov/pubmed/9354993. 10.1046/j.1365-2648.1997.00371.x 9354993

[12] Salaam S, Scott S, Richard SG, Heckathorn DD, Jarlais DCD (2009). Ethical and regulatory considerations in HIV prevention studies employing respondent-driven sampling. Int J Drug Policy.

[13] Goel, Salganik (2009). Respondent-driven sampling as Markov chain Monte Carlo. Statist Med.

[14] Heckathorn D (1997). Respondent-driven sampling: a new approach to the study of hidden populations. Soc Probl.

[15] Heckathorn D (2002). Respondent driven sampling II. Deriving valid population estimates from chain-referral samples of hidden populations. Soc Probl.

[16] Muraguri N, Tun W, Okal J, Broz D, Raymond HF, Kellogg T, Dadabhai S et al. Recruitment networks, HIV and STI prevalence, and risk factors among male sex workers and other men who have sex with men in Nairobi, Kenya. 2014. **(Manuscript submitted for publication)**. 10.1097/QAI.0000000000000368 PMC4973514 25501346

[17] Musyoki H, Kellogg TA, Geibel S, Muraguri N, Okal J, Raymond HF, Dadabhai S et al. Prevalence of HIV, sexually transmitted infections, and risk behaviours among female sex workers in Nairobi, Kenya: Results of a respondent driven sampling study. 2014. **(Manuscript submitted for publication)**. 10.1007/s10461-014-0919-4 PMC4786175 25428282

[18] Dita B, Okal J, Tun W, Sheehy M, Mutua H, Muraguri N, et al. High levels of bisexual behaviors among men who have sex with men in Nairobi, Kenya. Poster session presented at the International Aids Conference in Rome, Italy.

[19] Tun W, Sheehy M, Broz D, Okal J, Muraguri N, Raymond HF, Musyoki H et al. HIV and STI prevalence and injection behaviors among people who inject drugs in Nairobi: results from a 2011 bio-behavioral study using respondent-driven sampling. 2014. 10.1007/s10461-014-0936-3 PMC4352193 25398417

[20] Semaan S, Heckathorn DD, Des Jarlais DC, Garfein RS. Ethical considerations in surveys employing respondent-driven sampling. Am J Public Health. 2010;100(4):582–3. author reply 583–4. doi:10.2105/AJPH.2009.184200. 10.2105/AJPH.2009.184200 PMC2836345 20167881

[21] Kaplan C, Korf D, Sterk C (1987). Temporal and social contexts of heroin-using populations: an illustration of the snowball sampling technique. J Nerv Ment Dis.

[22] McKnight C, Des Jarlais D, Bramson H, Tower L, Abdul-Quader AS, Nemeth C, Heckathorn D (2006). Respondent-driven sampling in a study of drug users in New York city: notes from the field. J Urban Health Bull N Y Acad Med.

[23] Marpsata M, Razafindratsimab N (2010). Survey methods for hard-to-reach populations: introduction to the special issue. Methodol Innov Online.

